# Fertility preservation by ovarian tissue cryopreservation of children in China——umbilical single-incision surgery and perioperative experience

**DOI:** 10.3389/fendo.2025.1555189

**Published:** 2025-05-05

**Authors:** Zheng Qipeng, Liu Jing, Su Yun, Li Zexi, Cao Zhenhua, Chen Meng, Zhou Yan, Zhang Hua, Wang Zecheng, Tian Yu, Ruan Xiangyan, Wu Yurui

**Affiliations:** ^1^ Department of Thoracic Surgery and Surgical Oncology, Capital Center for Children’s Health, Capital Medical University, Beijing, China; ^2^ Department of Gynecological Endocrinology, Beijing Obstetrics and Gynecology Hospital, Capital Medical University, Beijing Maternal and Child Health Care Hospital, Beijing, China

**Keywords:** ovarian tissue cryopreservation, fertility preservation, surgical techniques, perioperative management, children

## Abstract

**Background:**

Ovarian tissue cryopreservation and transplantation (OTCT) is an effective method for preserving fertility and endocrine function. This study aims to summarize the surgical techniques and perioperative experiences to provide clinical evidence for pediatric OTCT.

**Methods:**

This retrospective study reviewed the clinical data of 89 children who underwent umbilical single-incision laparoscopic oophorectomy at Children’s Hospital, Capital Institute of Pediatrics between September 2020 and December 2024. The types of primary diseases were summarized, differences in preoperative complete blood count results, surgery methods and intraoperative conditions were explored among different primary diseases. Different surgical methods were reviewed. The surgery steps and techniques were summarized. The trends in surgical volume over time and the surgical learning curve were analyzed. The factors affecting follicle density were also explored.

**Results:**

The primary diseases in this study included Turner syndrome, aplastic anemia, mucopolysaccharidosis in, chronic active Epstein-Barr virus (EBV) infection, hematological malignancies, solid tumors, platelet dysfuction, metachromatic leukodystrophy, hemophagocytic syndrome, myelodysplastic syndrome, beta-thalassemia, osteopetrosis, dermatomyositis, congenital dyserythropoietic anemia, metachromatic leukodystrophy, and primary immunodeficiency. Children received chemotherapy will experience a decrease in white blood cell (WBC) and neutrophil levels, necessitating granulocyte-stimulating therapy; children with aplastic anemia had a significant drop in hemoglobin level, thus requiring red blood cell transfusions; children with myelodysplastic syndrome and aplastic anemia showed a marked decrease in platelet levels, necessitating platelet transfusions. Children with Turner syndrome most commonly have the unclosed internal inguinal ring. The main steps of umbilical single-incision laparoscopic oophorectomy were incision, trocar placement, observation, suspension, dissection, removal, and incision closure. The number of umbilical single-incision laparoscopic oophorectomy had been increasing year by year. The learning curve analysis indicated that the first 35 cases were the learning and improvement phase. Follicular density was significantly correlated with age, primary disease and ovarian color.

**Conclusion:**

Pediatric OTCT has broad applications and a promising future. Perioperative preparation and the surgical process are important. It is necessary to adjust the complete blood cell count to ensure that WBC greater than 4*10^9/L, neutrophils greater than 1*10^9/L, hemoglobin greater than 70 g/L, and platelet greater than 100*10^9/L before surgery. Given the small volume of children’s ovaries, it’s necessary to remove the entire ovary. Energy devices can be utilized, however, it’s essential to minimize mechanical, thermal damage, and warm ischemia time to the ovary, while also preserving surrounding tissues.

## 1 Introduction

The ovaries are the most critical reproductive and endocrine organs in females, responsible for maintaining fertility and the secretion of sex hormones. Premature ovarian insufficiency (POI) is defined as a decrease in ovarian function in women under the age of 40 (1). POI can lead to a reduction or loss of female fertility, accompanied by the premature onset of varying degrees of menopausal symptoms. New research suggests that the prevalence of POI is higher than previously estimated at 3.5% (2). Iatrogenic POI, which is related to healthcare practices, is a common cause of ovarian function decline, including surgery, radiotherapy, chemotherapy, and the intake of other ovarian toxic substances (3), accounting for approximately 50% of POI cases (4).

Ovarian tissue cryopreservation and transplantation (OTCT) involves the surgical removing part or all of the ovarian tissue from one ovary, or all of one plus part of the other before severe damage to ovarian function occurs. The tissue is processed into standardized thickness sections and preserved at low temperatures using biological methods. When the patient’s condition permits, the cryopreserved ovarian tissue is thawed and transplanted back into the body to restore ovarian reproductive and endocrine functions, effectively preventing and treating iatrogenic POI (5). OTCT protects fertility as well as endocrine function. It is the only fertility preservation method for patients with malignancies that cannot delay treatment and for prepubescent girls, and it is currently the most effective and promising method for preserving ovarian function and fertility (6).

In every aspect of OTCT, it is crucial to ensure the viability of the ovarian tissue. Surgical procedures play a vital role, and efforts should be made to minimize mechanical and thermal damage to the ovarian tissue and to reduce the duration of warm ischemia. Additionally, protection of surrounding tissues is also essential. This study retrospectively reviewed the clinical data of children who underwent ovariectomy surgery at our center for OTCT, summarizing the surgical techniques and perioperative experiences, to provide clinical evidence for the standardized management of pediatric OTCT.

## 2 Methods

### 2.1 Subjects

A retrospective review was conducted on children who underwent ovariectomy surgery at Children’s Hospital, Capital Institute of Pediatrics in Beijing, China, from September 2020 to December 2024. A total of 89 cases were included, and their basic information, perioperative clinical data were collected. This included preoperative complete blood count (white blood cell, granular leukocytes, hemoglobin, platelet, etc.), sex hormone (follicle-stimulating hormone, luteinizing hormone, estradiol, progesterone, prolactin, testosterone, anti-müllerian hormone), ultrasound examination results, surgery time, surgical procedures, hospital stay, and postoperative complications (hemorrhage, wound infection, etc.).

Preoperatively, informed consent was obtained from the legal guardians of all children, and the study was approved by the Ethics Committee of Children’s Hospital, Capital Institute of Pediatrics.

### 2.2 Preoperative assessment

Sex hormones and gynecological ultrasound examinations should be performed before surgery. Additionally, routine preoperative tests were performed, including complete blood count, biochemistry tests (hepatic function, renal function, ion levels, blood glucose, etc.), coagulation function tests, infectious disease markers (Hepatitis B virus, Hepatitis C virus, Syphilis, HIV), electrocardiogram, and chest X-ray.

Preoperatively, guardians were fully informed about the strategies for protecting fertility and ovarian endocrine function. The primary disease was evaluated as being in a stable condition that could tolerate general anesthesia for abdominal surgery. Ovarian function was evaluated, and the patients were assessed prior to obtaining the patients’ informed consent and agreement for OTCT at Beijing Obstetrics and Gynecology Hospital, Capital Medical University (Beijing, China).

### 2.3 Ovarian tissue sampling and transportation methods

All patients underwent laparoscopic surgery via a single umbilical incision. During the surgical procedure, efforts were made to avoid mechanical and thermal damage to the ovaries. Upon cessation of blood supply, the ovarian tissue was immediately transferred to sterile low-temperature transfer fluid. The tissue was transported at a low-temperature environment of 4-8 °C using a dedicated transport box to the Ovarian Tissue Cryopreservation Bank at Beijing Obstetrics and Gynecology Hospital (Beijing, China), within 24 hours, the methodology was the same as previous published articles (6–9). For children at high risk of ovarian malignancy, for example, solid malignant tumors located in the pelvic cavity, a small portion of the ovary cortex (2 mm^2^) was also taken for pathological examination to rule out the risk of metastasis.

### 2.4 Ovarian cryopreservation and quality assessment

Ovarian reserve can be assessed through transvaginal ultrasound follicle count, ovarian volume, AMH levels, age, and the density of primordial and primary follicles in the ovarian tissue (4, 10).

After the ovarian tissue had been transported to the Ovarian Tissue Cryopreservation Bank (Beijing, China), it was meticulously treated with a sterile surgical knife and forceps to remove the medulla, ensuring the cortex remained intact. The thickness of the processed ovarian tissue was approximately 1 mm, with each piece measuring about 4 mm by 8 mm. Subsequently, the worst - looking ovarian cortex slice via gross observation was stained with Calcein-AM (CaAM) (Sigma-Aldrich Chemie GmbH, Saint Louis Mo, USA), staining using a protocol published previously (8, 11). The analysis of the number of surviving primordial and primary follicles was conducted on standardized biopsied cortices. Circular cortical slices, each with a diameter of 2 mm, were harvested from various regions of the cortex, with each biopsy yielding a volume of 3.14 mm³ (6, 7). The follicle count per 2-mm biopsy was subjected to statistical analysis.

### 2.5 Statistical methods

SPSS 26.0 and GraphPad Prism 10.2.0 were used as statistical and graphing software. Age, hormone levels, complete blood count indicators, and other count data were expressed as mean ± standard deviation (for normally distributed data) or median and interquartile range (for non-normally distributed data), with the Shapiro-Wilk test used to determine data normality. Categorical data such as side, preoperative chemotherapy, and surgical complications were expressed as numbers and percentages. For comparing differences between two categorical data sets, t-tests or non-parametric tests were used. For comparing differences among multiple categorical data sets, analysis of variance was applied. For comparing differences between two or multiple count data sets, chi-square tests or Fisher’s exact test were used. The learning curve for a single surgeon was fitted using the cumulative sum (CUSUM) method, with the peak of the curve indicating the time to reach the peak. The correlation between age or ovarian maximum diameter and follicle density was analyzed using Pearson correlation. A p-value of less than 0.05 was considered statistically significant.

## 3 Results

### 3.1 Case overview

**Figure 1:**
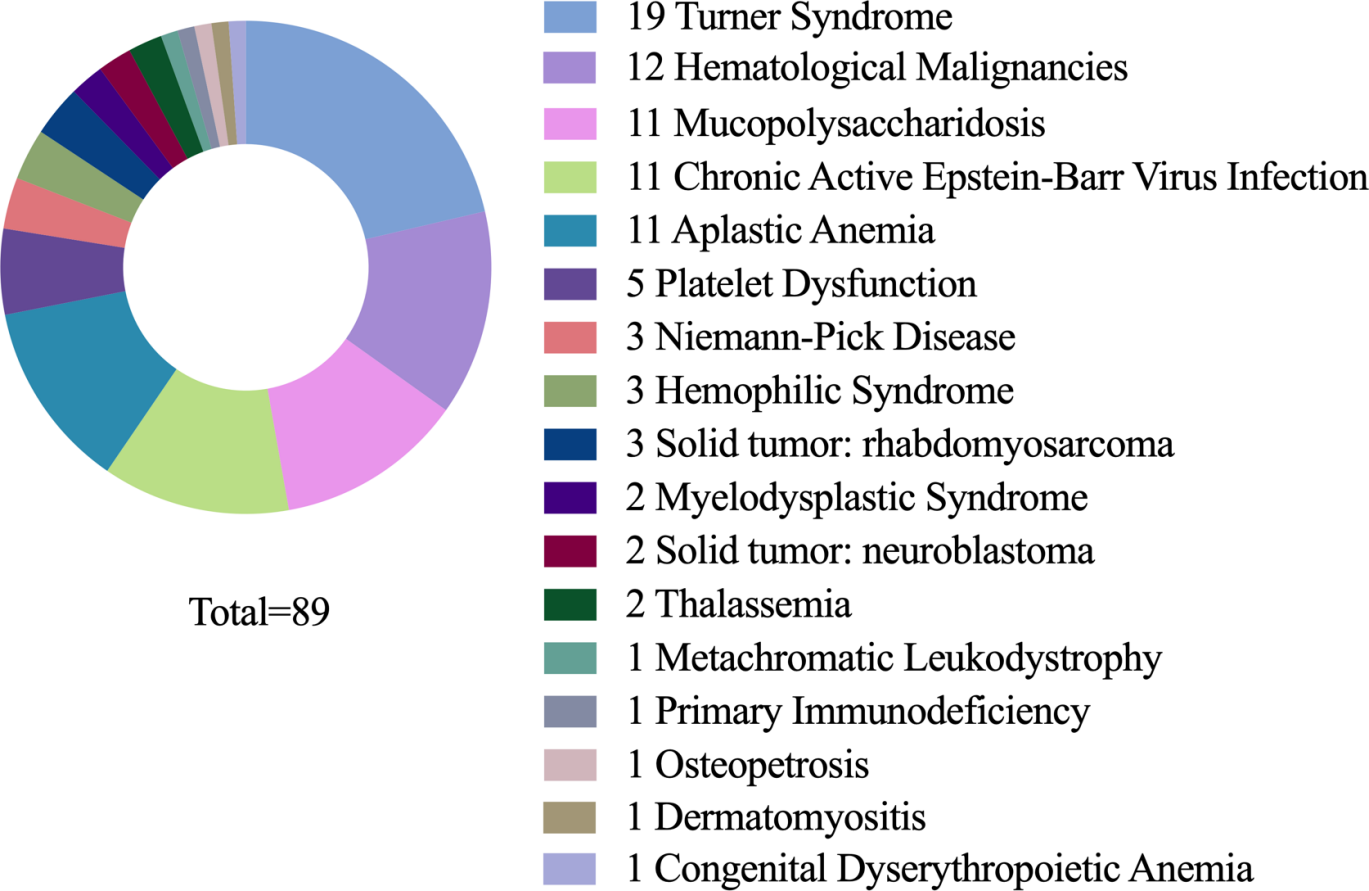
Distribution of primary diseases among study subjects.

A total of 89 eligible cases were included in this study. The primary diseases were distributed as follows: Turner syndrome (19 cases), aplastic anemia (12 cases), mucopolysaccharidosis (11 cases, with type I in 7, type IV in 3, and type VI in 1), chronic active Epstein-Barr virus (EBV) infection (11 cases), hematological malignancies (11 cases, including 3 cases of acute myeloid leukemia, 2 of Hodgkin’s lymphoma, 2 of acute lymphoblastic leukemia, 1 of NK/T-cell lymphoma, 1 of acute mixed-lineage leukemia, 1 of acute non-lymphocytic leukemia, and 1 of chronic neutrophilic leukemia), solid tumors (5 cases, with 3 cases of rhabdomyosarcoma and 2 of neuroblastoma), platelet dysfuction (5 cases), metachromatic leukodystrophy (3 cases), hemophagocytic syndrome (3 cases), myelodysplastic syndrome (2 cases), beta-thalassemia (2 cases), osteopetrosis (1 case, type 4), dermatomyositis (1 case), congenital dyserythropoietic anemia (1 case), metachromatic leukodystrophy (1 case), and primary immunodeficiency (1 case) (**Figure 1**). Additional information is presented in **Table 1**.

**Table 1:** Basic information of the study subjects.

Indexes	Results
Age (years)	7.76 ± 3.79
Prepuberty (<10 years)	63 (70.79)
Objective of ovarian tissue cryopreservation Hematopoietic stem cell transplantation Radiotherapy Anti-premature	64 (71.91) 5 (5.62) 20 (22.47)
Preoperative chemotherapy	24 (26.97)
Preoperative radiotherapy	0 (0)
Preoperative sex hormone levels FSH (IU/L) LH (IU/L) E2 (pg/ml) P (ng/ml) PRL (ng/ml) T (ng/dl) AMH (ng/ml)	3.09 (1.49, 5.67) 0.20 (0, 0.58) 15.54 (11.80, 33.96) 0.22 (0.14, 0.44) 10.08 (7.75, 18.19) 9.67 (6.86, 12.72) 1.22 (0.16, 2.65)
Preoperative complete blood count WBC (*10^9/L) Granular leukocytes (*10^9/L) Hemoglobin (g/L) Platelet (*10^9/L)	6.02 ± 4.04 2.13 (1.03, 3.45) 109.00 ± 21.30 233.80 ± 117.40
Operation time (min)	55.48 ± 23.13
Total hospital stay (d)	5.56 ± 1.27
Post-operation hospital stay (d)	2.24 ± 0.75
Ovarian cryopreservation sideways left right left+ partial right	16 (17.98) 71 (79.77) 2 (2.25)
Operative complication Hemorrhage Wound infection	1 (1.12) 3 (3.37)
Follicular count per 2-mm biopsy	468.20 ± 644.00

FSH, Follicle-Stimulating Hormone; LH, Luteinizing Hormone; E2, Estradiol; P, Progesterone; PRL, Prolactin; T, Testosterone; AMH, Anti-Müllerian Hormone; WBC, White Blood Cell.

### 3.2 Perioperative management

#### 3.2.1 Preoperative complete blood count

##### 3.2.1.1 WBC and neutrophils

**Figure 2:**
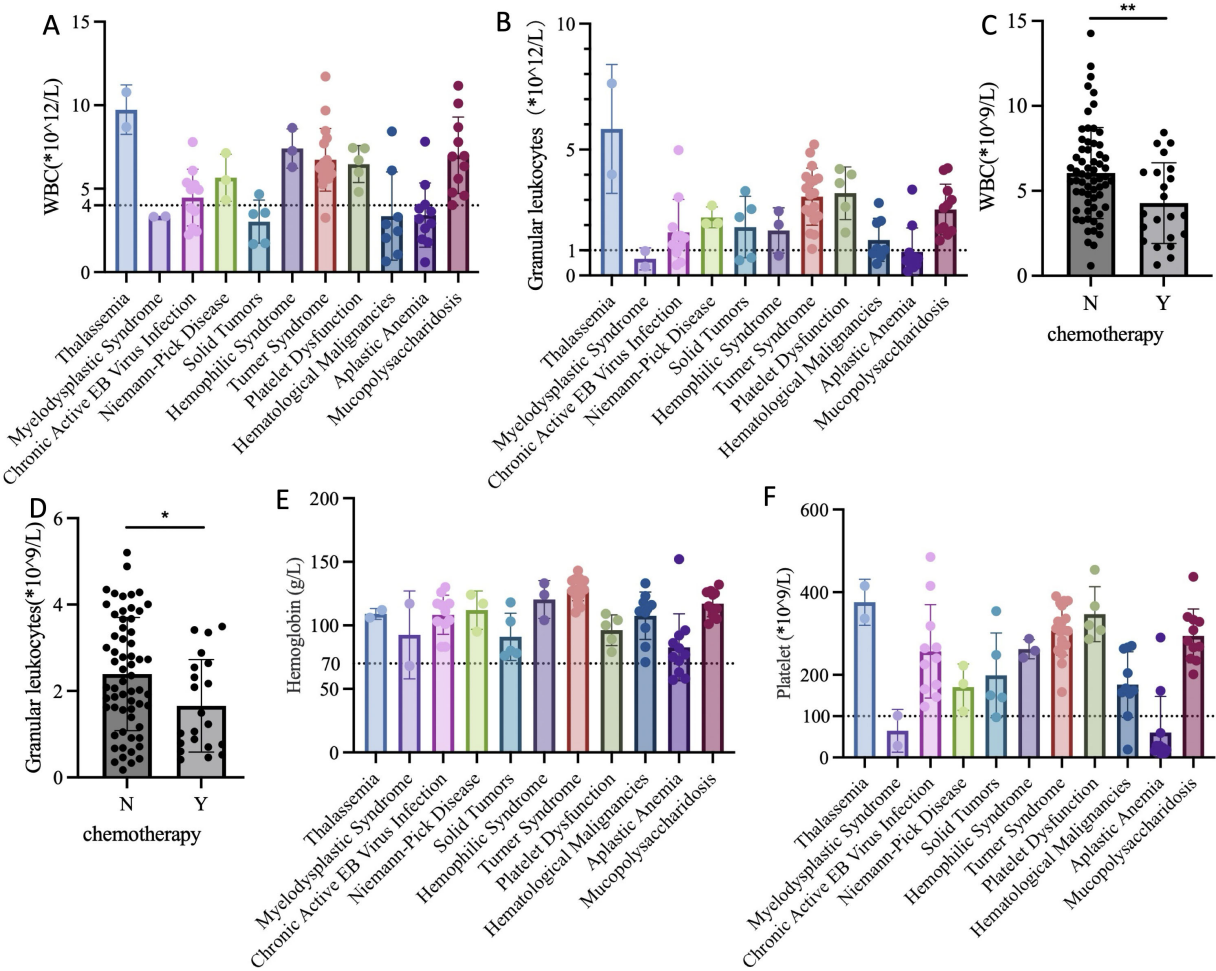
Preoperative complete blood count test for children in each group. **(A)** WBC levels of different primary diseases; **(B)** Neutrophils levels of different primary diseases; **(C)** WBC levels of chemotherapy and non-chemotherapy groups before surgery; **(D)** Neutrophils levels of chemotherapy and non-chemotherapy groups before surgery; **(E)** Hemoglobin levels of different primary diseases; **(F)** Platelet levels of different primary diseases. WBC, White Blood Cell; Y, Yes; N, No; “*”refers 0.01<P<0.05; “**” refers 0.001<P<0.01.

The results indicated that patients with myelodysplastic syndrome, solid malignant tumors, hematological malignancies, and aplastic anemia had significantly reduced WBC and neutrophil counts compared to those with other diseases (**Figures 2A, B**). Further analysis revealed that the decrease in WBC and neutrophils before surgery was closely related to the receipt of chemotherapy (P=0.007, P=0.024) (**Figures 2C, D**). Therefore, to minimize the risk of postoperative wound infection, they should have been administered granulocyte colony-stimulating factor subcutaneously. Surgery can be performed when WBC was greater than 4*10^9/L and neutrophils greater than 1*10^9/L.

##### 3.2.1.2 Hemoglobin

The results indicated that the average preoperative hemoglobin levels of children with different primary diseases were all above 70 g/L. However, some patients with aplastic anemia and myelodysplastic syndrome had levels below 70 g/L. These children received packed red blood cell transfusion before surgery. Post-transfusion complete blood count showed that hemoglobin levels were all above 70 g/L, meeting the surgical requirements (**Figure 2E**).

##### 3.2.1.3 Platelet

The results showed that children with myelodysplastic syndrome and aplastic anemia had significantly reduced platelet counts. To ensure surgical safety and minimize the risk of perioperative bleeding, platelet transfusion was required when platelet count was less than 50*10^9/L, or in cases of potential platelet dysfunction, such as thrombocytopathy. The optimal platelet concentration before surgery was greater than 100*10^9/L. However, considering the chronic shortage of platelets, the minimum platelet concentration required at our center was 50*10^9/L (**Figure 2F**).

#### 3.2.2 Surgical conditions

We endeavor to perform multiple invasive procedures and surgeries concurrently with laparoscopic oophorectomy. These include port implantation, bone marrow aspiration, and umbilical hernia repair. Moreover, any additional deformities or abnormalities detected during surgery are addressed simultaneously, such as unclosed internal inguinal ring, ovarian and fallopian tube cysts, and urachal anomalies. Our data reveals that out of 89 cases, 34 were found to have unclosed internal inguinal rings, with 14 exhibiting bilateral involvement. Children with Turner syndrome have the highest incidence of unclosed internal inguinal ring. Concurrently, 5 cases underwent umbilical hernia repair, 3 had port implantation, 5 had fallopian tube cystectomy, 2 had ovarian cystectomy, and 1 underwent urachal cystectomy followed by lymph node and liver tissue biopsy. Furthermore, for patients with malignant tumors that may involve the ovaries, a portion of the tissue was sent for pathological examination to exclude the presence of malignancy. In our cohort, two patients, one with pelvic rhabdomyosarcoma and one with hematological malignancy, underwent pathological examination due to the possibility of ovarian involvement, which was not ruled out preoperatively. The pathological reports confirmed the absence of malignant cell involvement.

### 3.3 Surgical techniques and key points

#### 3.3.1 Umbilical incision

**Figure 3:**
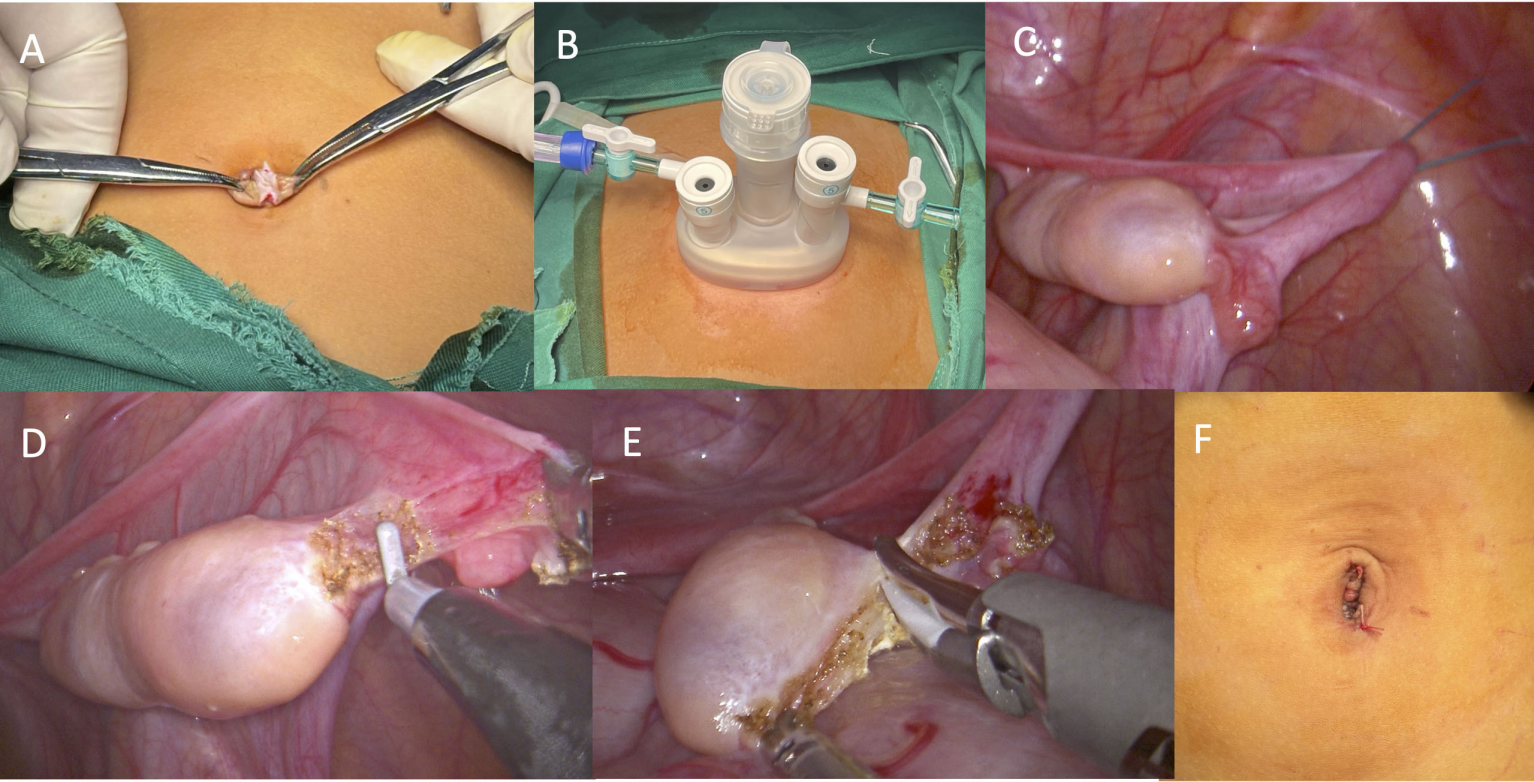
Surgical procedures. **(A)** A longitudinal incision at the umbilicus; **(B)** Insertion of a “disposable multi-channel single-port laparoscopic trocar”; **(C)** Suspension of the ovary and fallopian tubes; **(D)** The transection of the ovarian ligament; **(E)** The transection of the mesovarium; **(F)** Closure of the incision.

Make a 1–2 cm longitudinal incision at the umbilicus–, dissecting through layers until reaching the peritoneum, ensuring the skin incision does not extend beyond the umbilical ring (**Figure 3A**).

#### 3.3.2 Establishing surgical access

Insert a “disposable multi-channel single-port laparoscopic trocar”, connect to insufflation, and introduce the laparoscope and surgical instruments (**Figure 3B**).

#### 3.3.3 Exposure

Place the patient in a head-down, foot-up position. The surgeon stands at the head of the patient and observes the color, size, shape, and follicular condition on the surface of both ovaries. Select the optimal ovary, typically the one with a porcelain-white color, larger volume, full shape, and abundant surface follicles. If both ovaries are similar, choose the side that facilitates manipulation (usually the right side for right-handed individuals).

#### 3.3.4 Suspension

Penetrate the abdominal wall with 1–2 suspension sutures. The first suture should be positioned at the outer 1/5-1/4 of the ovarian mesentery. If manipulation remains difficult, a second suture may be placed near the infundibulopelvic ligament. The sutures should be applied close to the fallopian tube without causing damage. The suspended ovary, under gravity, allows the mesentery to fully expand, facilitating manipulation (**Figure 3C**).

#### 3.3.5 Dissection

In contrast to other surgeries, the dissection of the ovary is of paramount importance in ovarian tissue cryopreservation surgery. To maximize follicular viability, it is crucial to avoid thermal damage from energy devices and mechanical injury from surgical instruments. To increase the likelihood of conception in adulthood, the surgery must protect the fallopian tube fimbriae, the tube itself, and its blood supply to the greatest extent. Any accidental injury during surgery may lead to adhesions in this area, complicating future egg retrieval and transport.

The dissection should proceed from the distal to the proximal end, first transecting the ovarian suspensory ligament, and finally the ligament of the ovary. To prevent intraoperative and postoperative bleeding and to minimize ovarian warm ischemia time, electrocautery and ultrasonic scalpels may be used to dissect the ovary. However, the use of energy devices must be cautious, with single energy release times kept short, and energy release points should be more than 1 mm away from the ovary. Additionally, during the transection with an ultrasonic scalpel, the surrounding tissues should be protected using the clamp head of the scalpel (**Figures 3D, E**).

#### 3.3.6 Removal

To ensure follicular viability, it is essential to minimize the warm ischemia time of ovarian tissue. Therefore, before completely transecting the ovarian blood supply, the scrub nurse, surgical assistant, and ovarian tissue transport personnel should be prepared in advance to remove the ovary from the body and place it in a low-temperature transport box in the shortest possible time. The specific operation involves clamping a small amount of ovarian mesentery tissue with forceps, ensuring no damage to the ovary while maintaining a secure grip. Once the blood supply is completely transected, the ovary should be directly removed through the “disposable multi-channel single-port laparoscopic trocar” incision and quickly transferred to cryopreservation fluid for low-temperature storage.

#### 3.3.7 Layered closure of the incision

After the laparoscope re-examines the incision margin for active bleeding, close the incision layer by layer, resulting in a concealed and aesthetically pleasing postoperative incision (**Figure 3F**).

### 3.4 Surgical outcomes

#### 3.4.1 Annual increase in surgical volume

**Figure 4:**
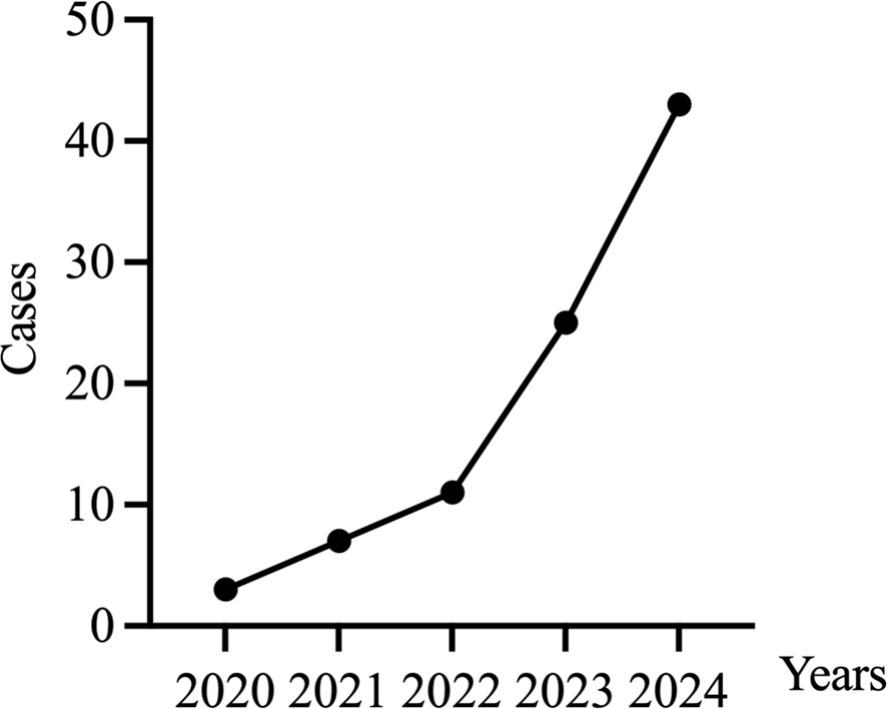
Annual surgical case volume.

The number of ovarian tissue cryopreservation surgeries at our center has been increasing annually, showing exponential growth. (**Figure 4**)

#### 3.4.2 Learning curve analysis

**Figure 5:**
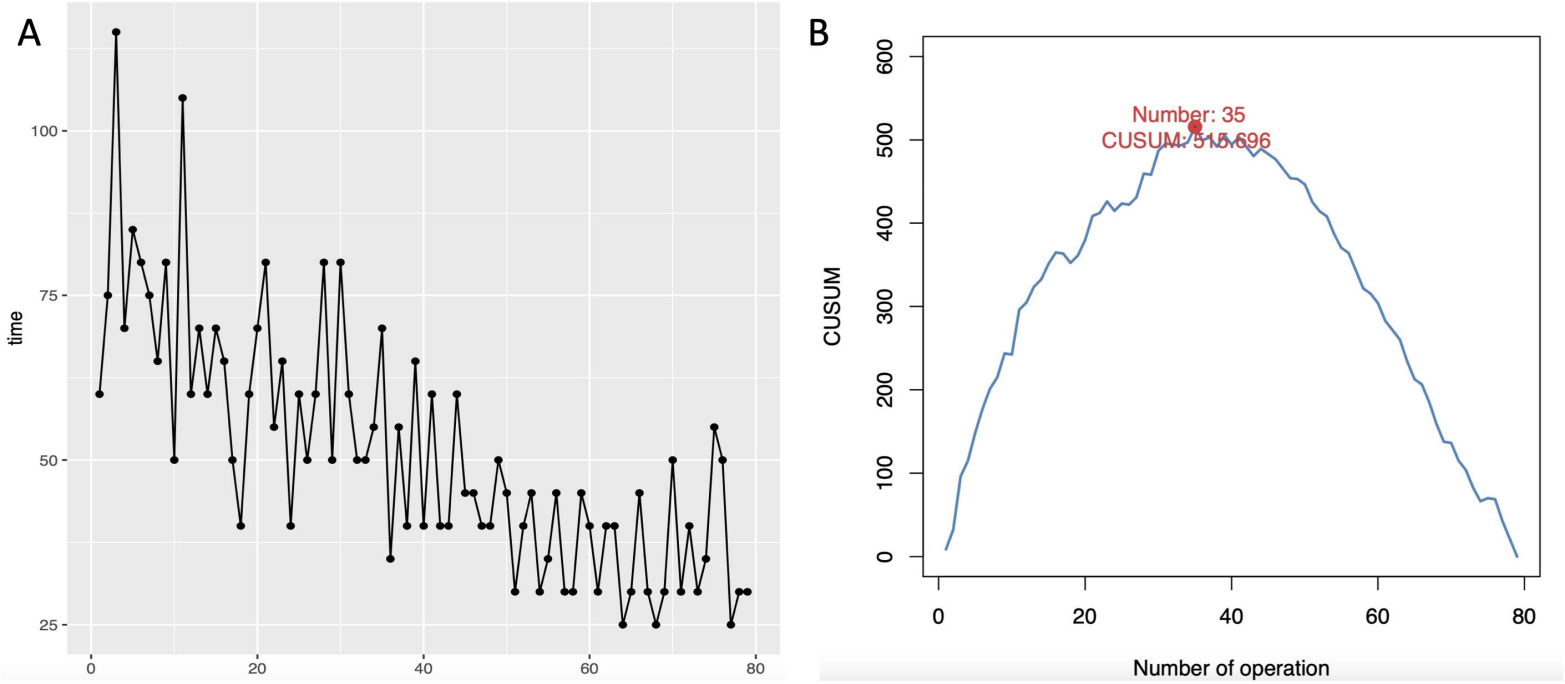
Learning curve analysis.

Since some patients undergo not only oophorectomy but also additional procedures or surgeries by a second surgeon during a single operation, these cases were excluded from the analysis of the surgical learning curve. A total of 79 pediatric patients were ultimately included in the learning curve analysis. All surgeries were successfully completed through a single-port umbilical approach, with no conversions to open laparotomy. As the number of surgeries increased, the surgical time gradually decreased. By plotting the learning curve scatter diagram using CUSUM values and fitting the equation (y = -0.482 + 29.826x - 0.505x^2 + 0.002x^3), the coefficient of determination (R^2 = 0.987) indicates a good fit. The peak number of cases was reached at the 35th case. Therefore, in this study, a single surgeon can be considered to be in the learning and improvement phase for the first 1–35 cases, and from the 36th case onwards, they are in the stable mastery phase (**Figure 5**).

### 3.5 Oocyte quality and quantity assessment

#### 3.5.1 CaAM results

CaAM is a non-fluorescent compound that freely permeates cell membranes. It is only converted into fluorescent calcein by lysosomal esterase in living cells, resulting in green fluorescent labeling. Our results showed good follicular viability across all ovarian tissues, as evidenced by strong CaAM staining, with no signs of compromised viability.

#### 3.5.2 Follicular density results

Follicular count per 2-mm biopsy was used to assess follicular density. The results demonstrated a significant correlation between follicular density and the primary disease. Notably, patients with Turner syndrome and myelodysplastic syndrome had very low follicular densities. Further analysis revealed negative correlation between follicular density and age (P=0.0027, R^2^ = 0.1058) and no clear correlation between follicular density chemotherapy treatment (t=0.2617, P=0.7942).

**Figure 6:**
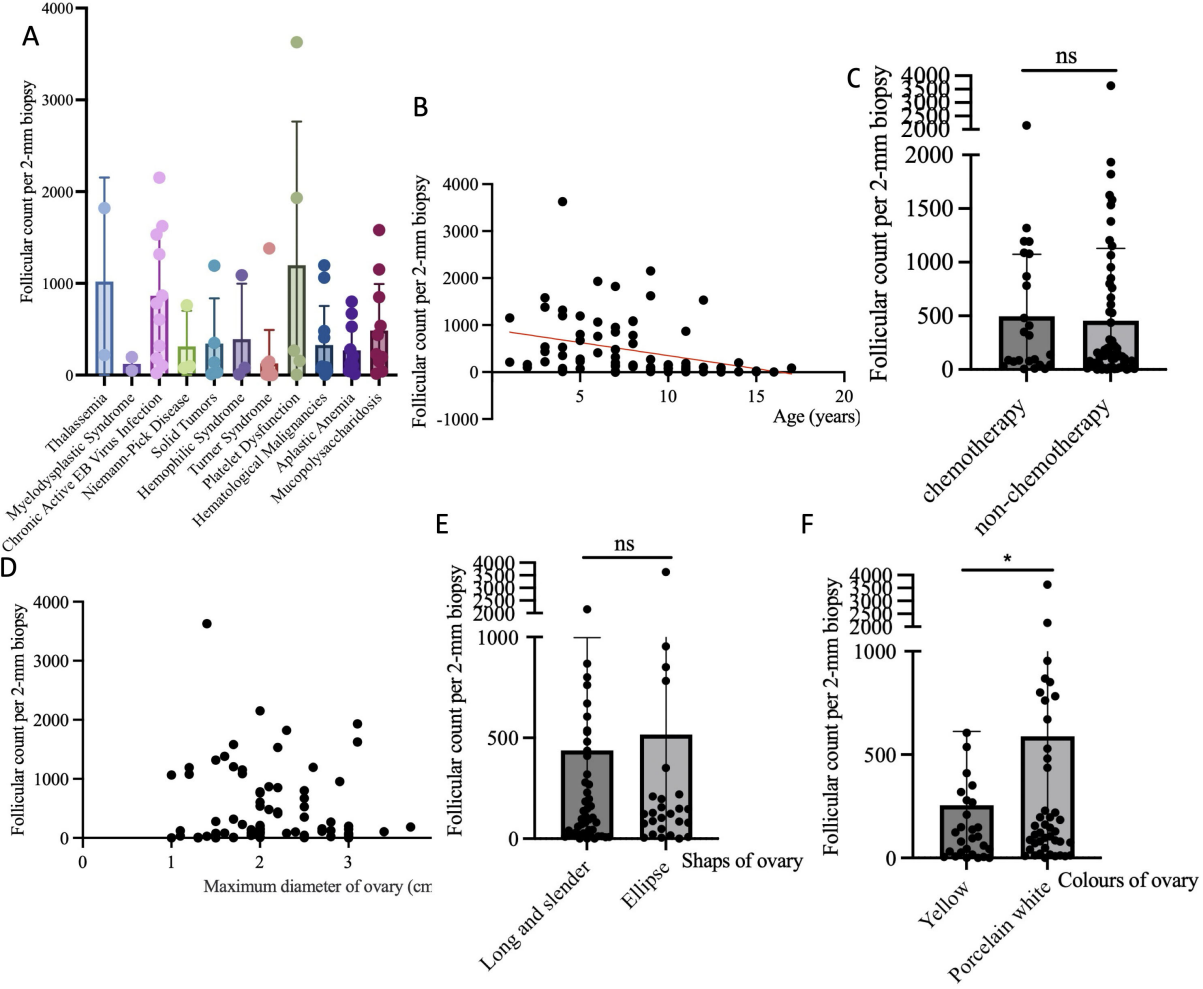
Analysis of factors affecting follicular density. **(A)** The correlation between follicle density and different primary diseases; **(B)** The correlation between follicle density and age; **(C)** The correlation between follicle density and chemotherapy before surgery; **(D)** The correlation between follicle density and the maximum diameter of ovary; **(E)** The correlation between follicle density and the shaps of ovary; **(F)** The correlation between follicle density and the colors of ovary. Notes: “ns” refers not significant; “*” refers 0.01<P<0.05.

We further analyzed the relationship between the intrinsic characteristics of the ovaries and follicular density. The results indicated no correlation between ovarian morphology and size and follicular density (P=0.5898; P=0.6439). However, ovaries with a yellowish color had lower follicular densities (t=2.330, P=0.0223) (**Figure 6**).

## 4 Discussion

POI refers to the decline in ovarian function before the age of 40 in women, with a global prevalence of approximately1%-3.5% (12, 13). Iatrogenic POI accounts for about 50% of cases (14), including radiation therapy, chemotherapy, surgery, and other intake of ovarian toxic substances. These treatments can cause irreversible damage to ovarian function. Additionally, the incidence of POI can reach 70%-100% following hematopoietic stem cell transplantation (HSCT) (15). OTCT is the most effective and promising method for preserving ovarian function and fertility in POI (16, 17).

Our team has been focusing on the theory and methodology of OTCT, leading to the establishment of the first Ovarian Cryopreservation Bank in China, as well as the development of a comprehensive set of guidelines and standardized operating procedures (6, 7, 18–20). We strictly control the indications, and our current technology and operational procedures are very mature, with two reported cases of successful pregnancy after transplantation (21, 22). As our technology continues to advance and the demand for fertility and endocrine function preservation grows among patients, the number of participants in our OTCT program increases each year. The range of diseases covered is extensive, including Turner syndrome, aplastic anemia, mucopolysaccharidosis, chronic active EB virus infection, hematological malignancies, solid tumors, platelet dysfunction, metachromatic leukodystrophy, hemophagocytic syndrome, myelodysplastic syndrome, thalassemia, osteopetrosis, dermatomyositis, congenital dyserythropoietic anemia, metachromatic leukodystrophy, primary immunodeficiency, etc. Notably, the proportion of Turner syndrome among these diseases is increasing, as children with Turner syndrome will develop POI. Children with Turner syndrome are at risk of developing POI, and previous treatment options were mainly centered around hormone therapy (23), which offered limited efficacy in protecting fertility and endocrine functions. OTCT has emerged as an effective solution to these challenges (24), and numerous clinical trials have been conducted internationally to confirm its effectiveness (25–27). In fact, an increasing number of scholars internationally are beginning to focus on the protection of fertility and endocrine functions in women, especially underage girls. The demand for OTCT has always been high, and with the technology becoming more mature, more patients are consulting and accepting OTCT, with the patient cohort becoming progressively younger (28, 29). In choosing patients for OTCT, we must be more rigorous in assessing eligibility. Preoperatively, we evaluate ovarian reserve by considering a host of indicators, including VMH, estrogen, progesterone, ultrasound, age, etc. As VMH hormone levels rise with age until 24, age becomes a key factor in our comprehensive assessment. Only patients who meet the criteria and have sufficient ovarian reserve are eligible for OTCT.

OTCT has been more extensively studied in adults, while the pediatric and adolescent population has received less attention (30). However, fertility protection is a crucial concern for underage female (31). In this study, all objects were children or adolescents. Among the 89 cases, 64 (71.91%) had to undergo HSCT due to various hematological diseases, a proportion consistent with reports (32). HSCT is a treatment method that uses high-dose radiotherapy or chemotherapy to eliminate tumor cells and abnormal clone cells in the body, while also destroying the patient’s immune system to reduce or eliminate the patient’s rejection of donor hematopoietic stem cells, and then infusing the patient’s own (autologous) or someone else’s (allogeneic) hematopoietic stem cells to rebuild normal hematopoiesis and immune function (33). The most common indications for HSCT are hematological diseases, including hematological malignancies such as leukemia, lymphoma, multiple myeloma, etc., as well as non-malignant hematological diseases like aplastic anemia, thalassemia, Fanconi anemia, etc. In addition, some congenital immunodeficiency diseases, hereditary bone marrow failure, and inborn errors of metabolism can also be cured through HSCT. HSCT conditioning not only kills the patient’s diseased cells and destroys the patient’s immune system but also causes irreversible damage to the ovaries, leading to a significant decrease or loss of fertility in adulthood, so OTCT is needed to protect ovarian function. For these children, the treatment of the primary disease is often urgent, and ovarian tissue needs to be cryopreserved before undergoing HSCT as soon as possible. Therefore, rapid recovery after surgery seems very important. Based on this, all children in this study underwent surgery through a single umbilical incision, which is both minimally invasive and aesthetically pleasing, allowing them to eat on the day of surgery and start ambulating the next day. Unlike adults, children have underdeveloped vaginas and hymens, making the transvaginal approach reported by Tetsuro Hanada (34) unsuitable. Thus, the single-umbilical-port approach is preferred. Single-umbilical-port surgery has many benefits for children. It causes less physical trauma, reducing postoperative pain and complications. It hides the incision in the umbilical area results in less noticeable scars and a more favorable cosmetic outcome. This also supports children’s psychological well-being. Additionally, this surgery shortens recovery time, enabling children to resume daily activities and school sooner. The “chopstick effect” in single-port surgery increases operational difficulty. However, as the number of surgical cases increases, surgeons will become more familiar with the procedures. Once they overcome initial operative challenges, they can master this operation.

The majority of children receiving OTCT treatment suffer from hematological diseases. Thus, adequate perioperative preparation can reduce the incidence of postoperative wound infections and other complications, ensuring a smooth transition to subsequent treatments. Preoperative assessment is crucial, including evaluation of complete blood count, hormone levels, and gynecological ultrasound. When there is a decrease in WBC, granulocytes, hemoglobin, platelets etc., corresponding treatment must be administered before surgery. Unfortunately, in our cases, three patients still experienced wound infection after surgery. The three children had hematological malignancies or solid tumors and had undergone chemotherapy before surgery, with varying degrees of bone marrow suppression, manifested by reduced WBC and granulocytes. Although there was a significant improvement after granulocyte stimulation treatment, the continuous effect of chemotherapy drugs could still lead to a delayed decrease in WBC and granulocytes, leading to infection. This also reminds us that for patients with reduced WBC and granulocytes before surgery, the wound healing situation needs special attention after surgery, and if necessary, the frequency of dressing changes can be appropriately increased. In addition, among the 89 cases we reported, two had malignant tumors as the primary disease, and a small part of the ovarian tissue was taken for pathological examination, with no evidence of tumor involvement. However, some studies have reported that a small part of the frozen ovarian tissue can detect malignant tumors (35), which also reminds us that pathological examination is necessary for patients with malignant tumors as the primary disease, especially pelvic malignant tumors. For those children, the complete ovary was resected, and a small portion of the proximal cortex, approximately 2 mm in size, was taken with a scalpel and immersed in formalin for pathological examination. Specific tumor-cell-specific immunohistochemical staining, corresponding to the type of primary disease, was performed to determine whether the ovarian tissue was affected by the tumor.

Currently, no studies have specifically reported on OTCT surgical techniques and precautions. Surgery is crucial for ensuring follicular viability in ovarian tissue cryopreservation. Unlike other surgeries, OTCT surgery must protect the ovaries and consider future transplantation effects. Thus, it has two main principles: minimizing thermal damage, mechanical injury, and warm ischemia time to the ovaries; and protecting surrounding fallopian tubes and blood supply to the fimbriae, laying the foundation for future ovarian function recovery after transplantation. Of course, the volume of the child’s ovaries is relatively small compared to adults, so the conventional approach is to take the entire ovary on one side. Studies have shown that taking a complete ovary on one side for cryopreservation does not increase the incidence of POI (36), and observational studies have found that menopause is only advanced by 1–2 years after the removal of one ovary (37). Animal experimental research has found that removing one ovary and half of the other ovary in rats does not affect the levels of estradiol and progesterone in the blood (38). This indicates that removing one ovary generally does not affect the normal function of the ovaries, so all our children have at least one complete ovary taken. Some studies support the reasonable use of energy devices in this situation, but they must follow the first principle mentioned above (39).

OTCT technology has currently reached a relatively mature stage; however, there is no standardized paradigm for surgical operations, especially for children, with various centers still exploring their experiences. Our center pioneered OTCT surgery for children in China. Initially, based on experimental theory, we avoided the use of energy devices in surgery. In the first nine cases, we employed a sharp dissection method using scissors to cut the mesentery. However, one child experienced intra-abdominal bleeding postoperatively, necessitating a second surgery for hemostasis. Since sharp dissection inevitably leads to oozing, which not only affects visibility but also increases the risk of postoperative adhesions and potential long-term effects on the function of the fallopian tubes and fimbriae. Additionally, the need to ligate severed vessels adds to the operation time. Therefore, we improved our surgical method to use electrocautery for dissecting the mesentery, performing this method in 10 cases. However, we found that when using electrocautery, tissues would shrink upon being cauterized, requiring additional sutures to increase the distance between the ovary and the surrounding fallopian tubes and fimbriae, thus adding extra surgical time. Consequently, we further refined our surgery to use an ultrasonic scalpel for dissecting the mesentery, which not only avoids damaging ovarian tissue but also minimizes surgery time, maintains a clear surgical field, and reduces postoperative adhesions. When using energy devices, it is particularly important to ensure that the energy release point is at least 1mm away from the ovary and fallopian tubes, requiring short, spaced, and multiple energy releases. Regardless of the surgical approach, we paid great attention to protecting the ovaries during surgery. After staining the ovarian cortex with CaAM, we found no impact on follicular viability in all 89 pediatric cases, which fully demonstrated the advantages of ultrasonic scalpel surgery. Endocrine function and fertility protection are long-term endeavors, and thus, every step in the process requires meticulous attention.

Analysis of the learning curve for a single surgeon in this study revealed that the first 35 cases were a learning and improvement phase, with subsequent cases entering a stable mastery phase. Since our center lacked initial industry standards for this procedure, continuous optimization and improvement of each surgical step and technique have been implemented. Considering the two significant surgical method evolutions, earlier adoption of the ultrasonic scalpel for mesentery dissection could have advanced the learning curve peak by approximately 15 cases.

Our research results indicate that follicular density was negative related to age and related to the primary disease, with Turner syndrome having the lowest follicular density among all diseases. This result was consistent with other report (32). Turner syndrome constitutes a significant proportion of our case composition, with these children being older, having poorer ovarian development, and lower follicular density. Interestingly, we found that follicular density is not related to the volume and shape of the ovaries but is related to the ovarian color, with yellowish ovaries having a lower internal follicular density. This result has certain guiding significance for clinical practice, suggesting that the color of the ovary is a very important reference when surgeons decide which ovary to remove.

In summary, the indications for pediatric OTCT are broad, and the application prospects are good, with surgery being a very important part of the process. Perioperative complete blood countmonitoring and adjustment are crucial for reducing surgical risks and postoperative complications. It is recommended, if possible, to adopt a single-port umbilical incision approach for surgery, adhering to two principles: minimizing ovarian thermal damage, mechanical injury, and warm ischemia time, and protecting surrounding normal structures. When removing a complete ovary on one side, using an ultrasonic scalpel can reduce bleeding and surgical time, but it is important to pay attention to protecting the ovary and its surrounding normal structures. Postoperative follicular density is closely related to the primary disease, age and the ovarian appearance color; therefore, during surgery, it is advisable to preferentially select the ovary that is whiter and larger in volume.

## Data Availability

The raw data supporting the conclusions of this article will be made available by the authors, without undue reservation.
